# "The Hospital" Nursing Mirror

**Published:** 1901-08-17

**Authors:** 


"The Hospital" Nursing Mirror.

Being the Nursing Section of "The Hospital."

[Contributions for this Section of "The Hospital" should be addressed to the Editor, "The Hospital" Nursing Mirror, 28 & 29 Southampton Street Strand, London, W.C.]

### Notes on News from the Nursing World.

### THE ROYAL RED CROSS.

The King has conferred the decoration of the Royal Red Cross on Miss Margaret Fanny Keyser and Miss Agnes Keyser, "in recognition of services rendered to the sick and wounded returned from South Africa."

### NURSES AT FOUR GUINEAS A DAY!

In consequence of a statement made in one of the daily papers that ladies were being sent out "to nurse the Boer women and children," with a remuneration of £4 4s. per day, several of our readers, moved by the natural desire of obtaining employment on such singularly generous conditions, have written to us for full particulars. We regret to destroy any hopes that may have been founded upon such an astounding statement, but, as we surmised, it is not accurate. The Secretary of State for War, to whom we applied for official information, advises us that our contemporary "is in error as regards the allowances to be given to nurses for the Boer women and children," and adds that, "the reference is probably to the commissions of ladies lately formed by the Government for the inspection of the Boer concentration camps." The appointment of this commission has already been mentioned in our columns, and though one of the ladies nominated is a trained nurse, her professional services are not engaged. The pay for nursing Boers is, in fact, the same as that for nursing our own sick, and practically the nurses are selected from the members of the Army Nursing Service Reserve.

### NURSES WANTED IN BRITISH COLUMBIA.

We have received from the correspondent at Victoria, British Columbia, who informed us that nurses are wanted in that colony, the following: "No nurse," she says, "should think of coming here without a midwifery certificate. Very often the patient might be from 10 to 30 miles from a doctor, and, as most of the private work here is maternity nursing, it is safer to hold a certificate for such. All surgical cases, rich and poor, go to the hospital. One district nurse is employed for the city, therefore there cannot be an opening in that branch. There are no homes for nurses in the city; each nurse has her own private rooms or lives with her family. The fees are for medical, surgical, and monthly nursing $15, or about £3 3s. per week; fever nursing from $20 per week. Travelling expenses from London to Victoria costs at least £30. Living is a little expensive. One room costs $6 per month without board. All the nurses usually board themselves when they are not at cases. The cost is about $16 or £3 5s. per month. One can dress much the same as in England. The best time for general nursing is from October till June. There is a great demand for maternity nurses all the year round, both in city and country. Massage treatment is in its infancy. Any nurse thinking of coming to British Columbia would have to take her chance of cases, and be entirely on her own responsibility." It will be gathered from our correspondent's statement, which has been delayed in consequence of illness, that the special need in British Columbia is for maternity nurses.

### THE MATRON OF THE SALISBURY HOSPITAL, RHODESIA.

Miss Georgina Ronaldson is now in London engaging nurses and probationers for the Salisbury Hospital, Rhodesia. In fact, she has almost completed her arrangements, but in case any of our readers may desire to communicate with her, we may state that letters intended for her should be addressed to 14 Oxford Terrace, Hyde Park, W. Miss Ronaldson, who was trained at the National Hospital for the Paralysed and Epileptic, Queen's Square, and the London Hospital, has been in South Africa for some years, and thoroughly understands the nursing requirements of the country. From 1895 to 1898 she was matron of Klerksdorp Hospital, and from December 1898 to March 1901 matron of the Alan Wilson Memorial Hospital, Buluwayo. Miss Ronaldson is a member of the Royal British Nurses' Association.

### NURSING IN BOMBAY.

There are some interesting details in the report of the committee of the St. George's Hospital Nursing Fund which we have just received from Bombay. The committee again express their entire satisfaction with the supervision of the nursing institution by the sisters of the All Saints Community and their gratification that the Order of Honorary Serving Sister of the order of St. John of Jerusalem was conferred on Sister Mary Edith for services rendered by the sisterhood in nursing the plague epidemics of 1897-1898. A similar honour was conferred on Miss Leavers, formerly of the Private Nursing Staff, and on Miss Parsons, a senior nurse in the hospital. The hospital staff now consists of 34 nurses. During the year 24 probationers were taken on trial, of whom 5 left "not being sufficiently strong for the work and life of a nurse," 4 were sent away because they were unsuitable, and 15 remained. On the private staff there are 18 nurses, who since January this year moved into their new quarters and greatly appreciate them. The furniture in the comfortable bungalow in which they are now housed has been defrayed by the contributions of the public, freely given. During last year the private staff nursed 234 cases, one of which—that of the little Prince of Udaipur—being of the unusual length of nine months. Twenty-four were midwifery and 5 were plague cases. One of the nurses formerly attached to the institution has joined the Army Nursing Service Reserve and is working in South Africa. The sister-in-charge states that the hospital staff has experienced great strain and undergone much hard work, owing to the patients in the men's wards having largely increased. There has not been a proportionate increase in the women's wards, but the task of nursing there is said to be more arduous owing to the closeness of the atmosphere and the heat. It had for some time been recognised that one nurse on night duty on each floor of the hospital was insufficient and in October last, the night watch was doubled, the second nurse being either a junior or probationer. This plan appears to have met all requirements.

### THE CURETON TESTIMONIAL FUND.

It is with much satisfaction that we refer to the movement for the presentation of a substantial testimonial, in the form of a sum of money, to Miss Cureton, the matron of Addenbrooke's Hospital, Cambridge. An influential committee, with the Mayor and Lady Jebb at the head, has been formed, and a number of subscriptions have already been paid or promised. We hope that the amount ultimately raised will be handsome enough to mark, in a manner that cannot be mistaken, the keen appreciation by Cambridgeshire people of the long and valuable services rendered by Miss Cureton. It is possible that some of our readers who would not otherwise have heard of the movement, may be glad to have the opportunity of identifying themselves with it. Contributions to the Cureton Testimonial Fund may be sent to Messrs. Barclay's Bank at Cambridge.

### MALE NURSES AND LICENCE DUTY.

Some of our readers have written to inquire whether as male servants they are liable to be taxed, and one of them informs us that he has been "interviewed by a representative from the Inland Revenue Department." Whatever the object of the representative may have been, there is no reason for doubt in the matter of a licence, for we have the authority of the Secretary of Inland Revenue for the statement that "the employment of a male nurse does not render the employer liable to male servant licence duty."

### A COMPROMISE AT NEWTON ABBOT.

The new phase of the nursing question at Newton Abbot—the alleged interference with the medical treatment of patients in the infirmary—has ended in a kind of compromise. The Guardians' Visiting Committee met in camera, and from their report it appears that the medical officer (Dr. Culross) produced copies of statements made to him by Miss Catherine Allen, containing particulars of a conversation between herself and Dr. Ley regarding the case of George Hammick, a child suffering from rickets; Miss Goodman, as to conversation with Dr. Ley, with reference to the case of Mrs. Moist, an old woman, who had had a fall; Miss Fisher, in regard to several cases in which Dr. Ley had made comments; and also from Miss Jeffery, the late superintendent nurse; and a letter from Nurse Haynes, stating that Dr. Ley had spoken to her about receiving another patient who, he considered, was suffering from suppressed measles. No direct evidence of interference by Dr. Ley was adduced, and, after much discussion, by 15 votes to 14 the committee came to the conclusion that "whilst being strongly of opinion that no guardian has interfered with the medical authority of the workhouse, the committee consider from the evidence placed before them that it is not sufficient to find that such interference has intentionally taken place." When this came before the general body of guardians it was passed, and the chairman of the committee (Mr. Hannaford) moved, "That the board inform Dr. Culross that they were perfectly satisfied with his conduct of the medical cases in the house, and also that they hoped he would have no further cause to complain of any interference from any member of the board." This was seconded by Dr. Ley, who once again declared that he had consistently supported the medical officer, and eventually the resolution was carried by 39 votes to 2. Like many other people, Mr. Burridge was unable to reconcile the form of the resolution which expressed the hope that the medical officer would have "no further cause to complain" with the report of the committee who were unable to find that interference had intentionally taken place. But "all's well that ends well," and the matter passes into history as another example of the queer sort of things done at Newton Abbot.

### LADY DUFFERIN'S ADMONITION TO BANGOR.

At the annual meeting of the Bangor District Nursing Society, the Marchioness of Dufferin, who presided, very wisely declined to take the view that because the nursing work had been satisfactorily done everything was perfect. It is true that the society has been able to pay the nurse, and to defray the incidental expenses, "but," as Lady Dufferin said, "that was all," and she affirmed that "this state of affairs should not be allowed to continue in a place like Bangor, where there are rows and rows of villas and large houses." She also pointed out that there is plenty of work in the district to employ the services of two nurses, and expressed her conviction that one cannot possibly attend to all the sick poor. As she so largely helps to maintain the society on its present footing, her gentle admonition may tend to wake up some of the Bangor people from their lethargy.

### LEARNING TO READ AT 50.

A working man was brought into a hospital ward one afternoon in a wheel chair. He was about 50 years' old, and had a neglected, uncared-for appearance and a sullen expression. He had come in for aneurysm in an advanced stage, and he was put into No. 6 bed; but it was obvious that there was no hope for him. Next day, the nurse, after having tried in vain to draw him out of his shell, took him a book, thinking he might like to read himself to sleep. "Take it away, I can't read," was the ungracious reply. "Hullo, do you hear that?" called out little Dick in the next bed; "isn't he an old silly? Why, I can read beautifully!" Dick was a pet. He had had pneumonia badly, but was up half the day now, and would leave shortly. "Shut up," growled No. 6, and drew his bed clothes over his head. Next day nurse was just going off duty when she heard a gruff voice call out "Nurse." "Well, No. 6," she said, cheerfully, "what do you want?" "Take a penny out of my locker and get me a spelling-book, will you? I want to learn how to read." The book was bought, and that night No. 6 had his first reading lesson. It was a laborious task, but he never tired of it, though he suffered a good deal of pain as the days went on. Little Dick became his favourite teacher, and it was a pathetic sight to see the dying man and the bright child poring over the book and spelling out the words. "I can read a sentence now," No. 6 told the doctor one morning as he went his round. Proudly and haltingly he read a few lines to the doctor. Poor No. 6, that was his last attempt—he died that night.

### SHORT ITEMS.

Miss Sarah Harper, of 38 Bolton Street, Lancaster, has left £500 to the Bury branch of Queen Victoria's Jubilee Institute for Nurses.—The s.s. *City of Cambridge* arrived from South Africa on the 7th inst. The following nursing sisters were on boad: Sisters Hutchison, Mazzacchi, and Grewer (on one month's leave). Two civilian nurses arrived on the 10th on board the s.s. *Lake Erie*, viz., Nurse A. E. Rogers and Nurse Gossage (time expired). Both are entitled to a free return passage to South Africa. The *Assaye*, which is due at Southampton August 26, has on board Nursing Sisters H. Hogarth and E. M. Sutton, who are invalids, and Nursing Sisters H. A. Lawrence, M. Roche, E. F. Upperby, B. L. Shaffers, and O. E. Barrow, on passage home.

### Lectures to Nurses on Medicine and Medical Nursing.

By Norman Dalton, M.D. London, F.R.C.P., Physician to King's College Hospital.

LECTURE XVII.—DISEASES OF THE ORGANS OF RESPIRATION—(*Continued from page* 235).

The Sputum (*Continued*)—*Bronchiectasis—Gangrene—Diphtheria—Phthisis.*

In cases of chronic bronchitis, and in certain other very chronic diseases of the lungs, it sometimes happens that a portion of one of the bronchial tubes becomes permanently dilated. This condition is called "bronchiectasis," which means expanded or dilated bronchus. In all cases of bronchiectasis there is a great deal of muco-purulent secretion manufactured by the bronchial tubes, and the power of coughing it up is diminished. Consequently there is a great difficulty in emptying the bronchiectasis, particularly if the dilatation consists in a bulging out of one side of the main tube. Hence we often find that the sputum is retained in the bronchiectasis for twenty-four hours, or even for two or three days. It goes on accumulating there until it is so abundant as to stretch the walls of the bronchiectasis, and then suddenly, by a strong cough, a large quantity of sputum—perhaps half a pint or more—is brought up all at once. This sudden expectoration of a large quantity of sputum is very characteristic of bronchiectasis, and it should always attract the attention of the nurse. The only condition which would simulate it would be the bursting into the lung of a large abscess in the liver or in the pleura (empyema). In these cases, however, the discharge of pus from the lungs would probably only occur on one occasion, while in the case of bronchiectasis it would be repeated at more or less regular intervals. If the pus came from the liver it might be bright yellow or green from the presence of bile, or it might be reddish-brown like chocolate if the abscess in the liver had followed dysentery, or it might contain hydatids, all of which appearances should be carefully looked for. Patients with bronchiectasis are often very uncomfortable when the dilated part of the tube is full of secretion and feel relieved when the large discharge of matter occurs. They sometimes discover that by assuming peculiar attitudes (such as lying on the bed and hanging their head and chest over the edge of it) they can succeed in coughing up the accumulated secretion. Any manœuvre of this kind will be noted by the nurse and should be carefully reported. The sputum is usually very offensive and is often thinner than the ordinary bronchitic secretion.

In cases of asthma the sputum has a pearly appearance and is expectorated in the form of a round pellet. It is not often seen in hospitals, as cases of asthma are not usually admitted unless bronchitis is present as well, and the sputum of the latter affection masks that of the former.

In some cases of cancer or sarcoma of the lung the sputum is rather characteristic. It is light pink in colour and gelatinous, and has been, not inappropriately, compared to red currant jelly.

The word "gangrene" means the death and putrefaction of any portion of the body, and it is very important for a nurse to be able to recognise the odour of gangrene. It is, of course, extremely offensive; but so are many other smells, and gangrenous material has a distinct smell of its own. It cannot be described, and nurses should therefore seek an opportunity of smelling it if they ever come across a case of gangrene. Occasionally the sputum is dark-coloured and has a distinct gangrenous odour, and as this indicates the presence of gangrene of the lungs the importance of recognising it is extreme. Gangrene of the lungs is usually a sequel to pneumonia, but it may also follow the impaction of an embolus in the lung when the embolus comes from a part of the body where putrefaction is going on—for example from the decaying bone in cases of old disease of the ear. Sometimes a very severe case of ordinary croupous pneumonia ends in gangrene of the inflamed part of the lung, but it is especially in cases of "insufflative" pneumonia that gangrene occurs. Insufflative pneumonia is sometimes called "aspiration" pneumonia or "deglutition" pneumonia, and is the variety of pneumonia which occurs when some foreign substance which is either septic or liable to become septic enters the bronchial tubes, and is not rapidly coughed up again. Not unfrequently the foreign substance is some small particle of food which has "gone the wrong way," either because the patient was unconscious while being fed, or because the muscles which enable him to swallow correctly were paralysed. But in other cases the foreign substance enters the bronchial tubes by ulceration. For instance, in many cases of cancer of the œsophagus the cancer spreads from the œsophagus to the trachea, and when ulceration occurs a communication is formed between the two tubes. The surface of the ulcer in the œsophagus is covered by foul putrefying material, and this is drawn into the trachea, through the perforation, at each inspiration and sucked up into the lungs, where it becomes the centre of a virulent inflammation which soon ends in gangrene. Even a needle or fish bone, if accidentally swallowed, may pass through the wall of the œsophagus into the lung and there set up a gangrenous pneumonia.

In cases of diphtheria pieces of the typical membrane are sometimes coughed up. They have been compared to pieces of washed leather. It sometimes happens that the formation of "membrane" in the bronchial tubes is so extensive that not only the trachea and main bronchi, but many of the middle-sized tubes are "lined" by it. As it soon ceases to be firmly attached to the wall of the tubes, it is often coughed up as one large mass, which, when floated out in water, unfolds, and is seen to be an exact reproduction or "hollow cast" of the trachea, main bronchi, and the chief branches of the latter. It looks like a regular branching tree, and should always be preserved. There is a very rare disease called "plastic bronchitis" in which a cast of the tubes exactly like the above is expectorated. It is very difficult to distinguish plastic bronchitis from diphtheritic bronchitis.

In "phthisis," or "consumption," the sputum often contains streaks of blood. Apart from this, it presents a very characteristic appearance, being expectorated in small soft round lumps about the size of a large pea. Each lump as it falls on the bottom of the spittoon flattens into a round mass like a coin, for which reason the sputum has been called "nummular," which means like money. The round flat masses do not flow together but remain distinct. Sometimes the sputum contains small yellow pieces which look like cheese. This cheesy or "caseous" stuff is the great characteristic of tuberculosis, and it is, of course, now universally known that consumption is tuberculosis of the lungs. The cheesy particles in the sputum are very important, as it is in them that the bacilli of tubercle are most easily found. There is nothing very special to be said about the nursing of cases of consumption, but anything like diarrhœa must be carefully reported, for diarrhœa is often the final complication which carries the patient off. It is due either to tubercles forming in the intestine or to lardaceous disease of the bowels. It is thought that the intestine often becomes infected by tubercle because patients swallow their sputum containing living tubercular bacilli. The latter escape death in the stomach and reach the intestine, where they settle and produce fresh tubercles. Patients must therefore be cautioned against swallowing their sputum. Another symptom which marks the beginning of the end in consumption is œdema of the feet. Cases of phthisis mostly die slowly, but there are two complications which may suddenly terminate the case. The one is a large hæmoptysis and the other the bursting of a tubercular cavity in the lung into the pleura. The former may cause death in a few minutes. If not, the only thing to do is to make the patient lie flat on his back and keep absolutely still. Ice may be given to suck and an ice-bag applied to the chest wall. If faintness set in, the foot of the bed must be raised and the legs bandaged from the feet to the thighs, so as to keep the blood as much as possible in the brain. It may be necessary to give brandy, but that remedy is liable to excite the heart and increase the hæmorrhage. If the doctor live far off, and the hæmorrhage continue, ten drops of laudanum may be given. The bursting of a cavity in the lung into the pleura admits air and pus into the latter, a condition called pyo-pneumothorax. It is usually fatal in a few days. The occurrence is marked by severe pain in the side, dyspnœa, and collapse. All that can be done is to give brandy and send for the doctor.

### A Visit to An American Sanatorium.

By an English Nurse.

So much interest is felt in the open-air treatment of consumption that possibly a short account of a sanatorium for this disease, situated on the outskirts of Boston, U.S.A., which I visited not long ago, may prove interesting.

### A Nurse Patient.

Like our own wealthier classes, who migrate abroad, the American with a chest usually winters down South, in such places as San Antonio, but it is only those with long purses who can do so. Hence such sanatoria as the one I visited. I had been asked by a friend who, herself sick, was unable to go, to convey some dainty to a nurse who was a patient there; so taking one of the innumerable electric cars, half an hour's ride brought me to Franklin Park, situated well outside the city. The sanatorium is a long two-storied building with what seems like two large conservatories attached. Having asked for the patient I wished to see, I was conducted up a stone staircase and along a corridor, and ushered into a particularly bright and sunny bedroom where, by the open window, I found the nurse sitting contentedly admiring the view. It was a beautiful one, for the window overlooked a large well-wooded stretch of open country, lying white and sparkling (for there had been a snowfall the day before) under the clearest and bluest of skies, whilst in the west the tallest trees stood up black against the crimson and orange of the sunset sky.

### Life in the Sanatorium.

Having delivered my message, I turned to the room. Its cleanness and whiteness fascinated me. On the whitewashed walls, as on the white paint of woodwork and flooring, there was not so much as a finger print. The snow outside was hardly whiter than the counterpane and covering of the easy chair, whilst the cushions of bright red gave the needed touch of colour. The patient told me of her life cheerfully enough. "Everything was comfortable—those who were unable to rise for breakfast had it brought. It was certainly tiresome at times if one wanted to close one's window to find one couldn't, and though it was pleasant for each patient to have a room, it would be nice to chat to one's neighbour sometimes. But, still, there was the sitting-room for that." I was much interested in the precautions taken against those who already suffered from the disease conveying it to others.

### The Precautions.

Each patient was provided with a small satchel containing a goodly supply of handkerchiefs made of rice paper; each carried also a pouch or bag, made of some pretty, cheap material, in which all used handkerchiefs had to be placed. These the nurse coming round at night collected and straightway put into the furnace. They also use an expectoration cup, which seemed to me much better than anything I have ever seen used in England. It is a square box of stout cardboard, made after the fashion one makes paper boxes for children. The cups are sold in dozens, folded flatly up; when unfolded they form the before-mentioned box, the top being formed of four flaps. This box fits into a wire casing, and at night these, too, are collected and burnt. It is very strictly enforced on all consumptive patients that for the public weal they must be careful in these matters, and thus the danger of inhaling germs liberated from dried sputa is lessened, and the nurse is spared the risk of washing out infected vessels.

### The Patients' Rooms.

However, to return to the sanatorium itself. I found that the women had their rooms on the upper floor, the men on the lower, all patients' bedrooms having a southern aspect. At one end was the sitting or sun-room, into which through glass roof and sides sunshine and warmed fresh air poured all day; the room itself looking a picture of comfort with its lounges and reading tables, books, papers, and flowers. Here we found several patients, girls mostly, talking brightly. The arrangement of the chapel was rather funny. The men entering from their own floor occupied the body of the chapel whilst the women entering from above occupied a kind of gallery. Throughout the entire building the keynote was cleanness and simplicity, which yet escaped bareness. As we passed a door near the dining room, my guide told me that an English nurse was there whom she was sure would like to see me.

### Do You Know Guy's?

Having obtained the necessary permission, I knocked, and in answer to a cheery "Come," went in. On the pillow lay one of the brightest and merriest, and, I think, thinnest faces I have ever seen. For a minute an attack of coughing prevented the patient speaking, and a lump came into my own throat; it seemed so sad and hard that she should be lying dying alone, among strangers, so far from friends and country. When she recovered breath enough to ask questions her first query was, "Has London changed much? and oh! do you know Guy's?" For half an hour I poured out all the information I could as to what had changed, what remained the same, in great London, and in our little nursing world, till fearing to overtax her strength, in spite of protests, I left her, promising to come again.

### Her Story.

"She is just the cheeriest, pluckiest woman I ever came across," the superintendent remarked as he courteously explained how, in spite of twenty degrees of frost outside and every window being wide open, the entire building was kept at so pleasant a temperature. "Why, she is quite alone. She lost husband and children (four of them) within a year, I believe, then returning to nursing she worked on bravely and brightly till disease had her fairly in its grip." I learnt afterwards that she died shortly after my visit, bright and cherry till the last, and thinking it over I came to the conclusion that one might do worse than end one's days in that lovely spot surrounded by such care and kindness. To the sick English stranger, at any rate, our American cousins show themselves both kin and kind.

### Free to All.

As regards the nurse first mentioned, I heard a few weeks ago that she had been discharged cured. I may add that this sanatorium is free to all. I believe, but am not quite certain, that the City of Boston contributes partially towards its support, whilst private liberality and benefactions do the rest. The percentage of recoveries the superintendent informed me was good, considering the fact that patients seldom arrive in the earlier stages of the disease. I may add that windows are provided with wire shades for use in case of wet weather, which whilst admitting air keep the rain out.

### To Nurses.

We invite contributions from any of our readers, and shall be glad to pay for "Notes on News from the Nursing World," or for articles describing nursing experiences, or dealing with any nursing question from an original point of view. The minimum payment for contributions is 5s., but we welcome interesting contributions of a column, or a page, in length. It may be added that notices of entertainments, presentations, and deaths are not paid for, but, of course, we are always glad to receive them. All rejected manuscripts are returned in due course, and all payments for manuscripts used are made as early as possible after the beginning of each quarter.

### Lectures to Head Sisters.

By E. MARGARET FOX, Matron of Tottenham Hospital.

LECTURE II. (*continued from page* 253).

ON WARD MANAGEMENT.

A HEAD sister must never consider the constant inspection of lockers beneath her, or she will find a varied assortment of the things that should not be, ever growing. They must be systematically turned out twice a week after visiting hours, and all contraband goods watched for. Either the evening of visiting day, or the next morning is a good time for doing this. Rules, of course, vary in different hospitals as to what patients may have brought in to them by their friends, but generally speaking, no eatables of any kind should be kept in lockers, no money beyond a few pence, no clothes beyond a clean shirt, towel and handkerchief, and no ink.

If you value spotless bed linen you will absolutely prohibit the use of ink by patients in bed. Spectacles, slippers, note-paper, and a book or two, are permissible. In the children's ward, you must not let the lockers be filled with toys, and when the rules allow of eggs, oranges, biscuits or sponge cakes being brought in, have them all kept in the ward kitchen, and mark the eggs with the patients' names. Also, be sure to make the friends of the children understand that cakes, etc., will be kept in a common tin and shared among such of them as are allowed to have them, at lunch and supper.

Give out dressings and bandages yourself from the cupboard as required, and exercise strict supervision over the way the stores are used, remembering that in the hands of an inexperienced probationer such things as iodoform gauze, and absorbent wool are often cut very much to waste, a great deal more being used than is needed. Ordinary cotton wool costs less and does quite as well as absorbent for covering fomentations and ointment dressings, and for extra bits of padding for splints—in fact anywhere where absorbent qualities are not needed.

When you have a patient who is likely to be on fomentations for some time, it is a good plan to cut as many pieces as will be required in the twenty-four hours, at once, and not leave an open packet of boracic lint or wool, to be torn off by anyone as needed. Keep two bandages in use for a fomentation, one being ready rolled in the locker, so that there is no delay when applying the dressing, which often results in a new one being used each time to avoid waiting. Try to impress upon those working under you two things in connection with the use of dressings—the necessity for economy in dealing with expensive items such as iodoform gauze, gutta-percha tissue, and jaconet, and the absolute necessity of cleanliness in touching them. Wool and gauze must only be cut by most carefully washed hands, and should be laid on a clean towel, and in every way protected from contact with dust or dirt. If called away in the middle of cutting it, see that it is covered up, and not left exposed to the air. It should never be cut while sweeping and dusting is going on in the ward.

Absorbent wool goes further, if aired before being used, and should be hung on a clean towel, not too close to the fire, as it scorches so quickly. This sort of thing is best done in the evening, and if you have a busy ward, you will also find a perceptible lightening of your morning labours, by having your dressing waggon and test table cleaned over night, and covered up to preserve the things from dust.

Never allow uncovered specimen glasses of urine to remain in your ward all night, any more than you would allow bed-pans. If they must be saved till the next day, keep them in the lavatory, and see that they are covered.

Bed-pans and urinals should be given round at stated times by the junior probationer when on duty, or the senior when she is on. Be careful that they are always given and removed with the covers supplied, and that they are never put on the floor or lockers. Of course, absolute laws cannot be laid down about giving them out, as one must not be refused to a patient who asks for it; but only by the exercise of tact, you must try to avoid having them given at inconvenient moments.

Beds should also be tidied regularly at certain fixed times. If they are made by the night nurse, they will require straightening about ten o'clock, after the patients are washed, and bed-pans and lunches given. Tidy them again after each meal, and before you go off duty in the evening, see that the sheets are evenly turned down and the quilts straight.

Have the bedsteads well carbolised after a death occurs or when a patient goes home. Mattresses require baking after a doubtful case, or a death, and should be frequently brushed to get rid of the dust that will collect in spite of all efforts. Have one day in the week for scrubbing mackintoshes in turn, and keep those not in use rolled and not folded, as they wear so much longer.

New blankets should not be used for cases that are dying, nor for typhoids, nor dirty new admissions. Blankets begin to depreciate in value as soon as they are washed, so they should be kept clean as long as possible.

Sheets must be regularly changed, the top sheet being always put for the bottom one, and a clean one on the top. Pillow cases should be protected by towels or mackintoshes in all mouth and head cases. Clean quilts should be put on those beds farthest from the fire first. And when a new case is being washed, see that the rug or blanket on which he lies reaches well up over the pillows, or his dirty head will necessitate another clean pillow-case before he is finished. A towel should always be tucked under a patient's chin after an operation, in case of sickness, and a cloth or bed-table should be used for meals, to prevent the sheets getting stained and greasy. Good management will make the supply of ward-linen go twice as far, and the beds will always look clean and tidy. In a women's ward the patients will generally be glad to do any little necessary mending, and it is inexcusable to allow buttons and tapes to be missing day after day. Jackets, and if possible gowns also, should be changed at night, and always aired before being put on again. Clean gowns will be needed twice a week, if not oftener.

You can largely control waste in the drug department by looking personally after the medicines and lotions used in your ward. Go through your medicine cupboard once a week, retaining nothing but stock things there, or you will find a heterogeneous collection of bottles and jars will grow with mushroom rapidity. Many drugs spoil by keeping, which could be used if sent back to the dispensary as soon as done with. When a medicine is likely to be changed, keep the empty bottle back until after the doctor's visit, so that eight or ten doses of a fresh bottle do not have to be thrown away. See that the medicines are given regularly, punctually, and in a tidy, cleanly manner. Never forget to read the label and shake the bottle, and when pouring it out to keep the label uppermost. You thus avoid soiling it, and cannot help reading the directions. The accurate giving of medicines is more important than a great many nurses seem to think, and to ensure this accuracy, the keeping and daily revising of a medicine list is very necessary, and should not be neglected, even though only one patient happens to be taking a medicine at the time.

### Action for Libel by a Hospital Nurse.

REMARKABLE EVIDENCE.

At the Leeds Assizes last week the case of Martin *v.* Allen was heard before the Lord Chief Justice. This was an action for libel brought by Miss Elizabeth Martin, described as a certified hospital nurse, residing at 262 Glossop Road, Sheffield, against Richard Grammar Allen, surgeon, of Belper. Plaintiff claimed for £500 damages. The action was commenced in consequence of a letter sent by defendant to the Belper Isolation Hospital Committee, on July 11th, 1900, in which certain allegations were made by him against the plaintiff reflecting on the management of the hospital.

Mr. Tindal Atkinson said the plaintiff held high diplomas for medical and surgical nursing, and also for midwifery. She was appointed matron of the Belper Isolation Hospital in May, 1897. She found the hospital in anything but a satisfactory state, and she took steps to put things on a more satisfactory basis. Later on the defendant was appointed medical superintendent, and from the first there had been friction between him and the plaintiff. On more than one occasion the plaintiff had to remonstrate with the doctor as to the way he went about his work. On June 18th, 1900, she complained to him about winking at one of the nurses, and on the 11th of the following month he wrote a letter to the committee, in which he made grave charges against the plaintiff. Counsel read the letter, which stated that plaintiff appeared to think the building was erected for her sole use and benefit as a convenient centre for friendly visitation and the dissemination of idle gossip; that the nurses had been worried by her petty annoyances; that she had made the position of the nurses of the hospital unbearable; that her manner to him had been insolent; that she practically monopolised the ambulance; that she had allowed the place to be turned into a show ground for the parents and friends of the patients, and that nurses would not come to the hospital from the Derby Nurses' Home as long as she was at the hospital. It was impossible, said counsel, for plaintiff to sit under such charges. She was asked for and gave to the committee her reply, whereupon she was dismissed. The defence of privilege and justification had been set up, but if it was shown that the letter was written from some indirect motive in consequence of matters that had occurred between the parties the defence of privilege would not avail, and the other side would have to rely on the plea of justification, that the charges were true in substance and fact.

### Plaintiff's Evidence.

Miss Martin stated that she had got diplomas for nursing from the Royal Nursing Institution, and also for midwifery from the Royal Obstetrical Society. She said that when she went to the Belper Hospital Dr. Galer was the medical superintendent. Afterwards Dr. M'Elligott held the post, and he was succeeded by the defendant. After May 20th relations between witness and the defendant became somewhat strained. On May 11th she spoke to him about leaving a message with a probationer when she herself was present, and later on she had occasion to speak to him because he gave a signal to one of the nurses in the wards.

The Lord Chief Justice: What sort of a signal?

Witness: He winked his eye at her and she left the ward and went out into the grounds.

Continuing, plaintiff said she objected very much to his behaviour in the wards in the presence of the patients. She denied in detail that all the allegations in defendant's letter were true.

In cross-examination Miss Martin admitted that previous to Dr. Allen coming to the hospital there was a slight grievance between Dr. M'Elligott and herself. On May 9th Dr. Allen asked her what this grievance was. He said he had often heard bits of gossip in Belper and he would really like to hear for himself. She told him it was a very unpleasant thing, but Dr. Allen persisted, and eventually she told him that it was about Dr. Galer and his housekeeper. Subsequently she said that eight nurses resigned during the last six months that she was at the hospital, but they did not all tell her why they left, and at any rate she did not think they resigned on her account. She denied that she gave them any annoyance. She believed that it was beneficial to the hospital that five of them did resign, although the other three were good nurses. She admitted that it looked peculiar that all of them should resign as they did, and if she did not know the circumstances it would appear peculiar to her.

In reply to counsel for defendant, Miss Martin said that she suggested that Dr. Allen was the author of a plot against her.

George Preston, journalist, of Alfreton, a member of the Hospital Committee, said he thought the management of the hospital was satisfactory, and that plaintiff did her work well.

Dr. Hooper, Job Spencer, Joseph Bown, and John Booth, all members of the committee, gave similar evidence.

John Stones, a porter at the hospital, said he had never seen an undue number of visitors in the grounds.

Evidence in plaintiff's favour was also given by Mrs. Cooper and Miss Olive Smith, who had been employed at the hospital.

### The Defence.

Opening the case for the defence, Mr. Scott Fox said there had been two or three doctors in charge of the hospital, and Dr. Allen was only appointed in January, 1900. As medical superintendent he was really responsible for the efficient management of the hospital, and it was his duty to exercise the utmost freedom and confidence in the reports he made to the committee, to which he was responsible. Counsel thought the jury would conclude that such an occasion was privileged. On January 10th, 1900, when his predecessor resigned, Dr. Allen was informed that another matron had been appointed.

The Lord Chief Justice: We should not have heard that. It may be at the root of the whole business. You are not entitled to open with any communication made to him regarding plaintiff.

Counsel went on to say that notice was given to plaintiff determining her engagement, and she also sent in her resignation, but she was afterwards allowed to withdraw it, and Dr. Allen said he would have no objection to work with her. Nurses resigned, and Dr. Allen found that Miss Martin's conduct was not such as made the administration of the hospital comfortable for the nurses. Two other nurses resigned in April, and letters of complaint were written by nurses who had complained to Mr. Pym, Clerk to the Hospital

Committee, and had been told by him they had better put their complaints into writing. Dr. Allen also was asked by Mr. Pym to put his statements into writing, and did so. He would ask the jury to say, after hearing the evidence he would call, that upon the information before him defendant was justified in the communication he made.

### Dr. Allen's Evidence.

The defendant, Dr. Allen, of Belper, said he held the post of medical superintendent to the Isolation Hospital on a retaining salary of £35, which might rise by fees to a maximum of £75 per annum. Just after his appointment he had an interview with Miss Martin, and expressed the hope that they would get on well together. At the end of January, the three nurses left at twenty-four hours' notice, saying they could not work with Miss Martin. It was untrue that he had winked at Nurse Sykes, or that he met her out in the garden. New nurses were appointed, but two left at the end of April, one saying she could not work with the matron; and in place of these Nurses Perkins and Sykes were appointed, and remained until the end of July. About May plaintiff's conduct was very impertinent towards him.

Witness went on to say that he spoke to plaintiff about going out in the ambulance, as he thought she ought not to go. On June 2nd he was at the hospital. It was visiting day, and he saw the visitors, instead of being at a window, were all over the place. He had been told it was no use applying to the Derby Nursing Institution for emergency nurses as they would not be sent to work under Miss Martin. Nurses Perkins, Sykes, and Kean all began to complain to him about the matron shortly after appointment, and witness added, in reply to the Judge, that he formed the opinion that the complaints were well founded, and that Miss Martin was unfit for her position. He had not the slightest ill-feeling against her on any account. He had not, either directly or indirecfly, requested the nurses to complain. He still believed, as he did when he wrote, that the statements in his letter were true.

In the course of his cross-examination Dr. Allen said he did not think the gate-book was honestly kept, because plaintiff did not want people to know when she went out and returned.

Mr. Atkinson: On one occasion she had gone to Derby in the ambulance, when it was going to be repaired, instead of having a carriage. Do you make that a charge against her?—Yes.

Witness went on to say that the promiscuous mingling of patients and visitors would destroy the whole advantage of an isolation hospital. He could not recollect that he had complained of this at once.

Mr. Atkinson then read this passage from the letter:—"Such a rabble I never came across . . . wandering here, there, and everywhere over the grounds. The matron's sole idea seems to be to make the place into a show ground to impress parents and friends. All this is exceedingly nice, but one or two have returned as patients."

Witness could not remember who had returned as patients after visiting, but he knew they did. He got it through some of the nurses. It was also a nurse who told him that a girl's dress was returned to her after being altered by a patient without being disinfected, and he believed she caught the disease through that.

Witness did not accept very readily Mr. Atkinson's suggestion that Nurse Sykes was rather a pretty nurse. She was passable, he said, but he denied all suggestions that he winked at or met her in the garden. He was not prepared to deny the sworn statement that the woman who went to do washing changed her dress before leaving. Had he known Miss Martin was not leaving he doubted whether he would have accepted the appointment.

### The Winking Incident.

Mrs. Ellen Spencer, of Belper, who was an inmate of the hospital for several weeks, gave evidence as to a woman who went as a visitor afterwards returning as a patient. She had seen the laundry-woman both morning and evening, and did not think she had changed her clothes. In cross-examination regarding a letter to a newspaper, over the signature "An Adult Patient," in which complaints were made about the matron, witness said Nurses Perkins and Kean asked her if they could put in the statements that were made. One of them wrote the letter, and witness saw and approved of it.

Mrs. Lucy Priestley, formerly Nurse Sykes, emphatically denied the winking incident. She complained of Miss Martin's treatment, and wrote her letter without any other person's suggestion except Mr. Pym's. She had wanted to wear caps with strings to them, but Miss Martin said the strings would provide a refuge for germs.

Mr. Atkinson: And you never liked her after?—I never liked her before.

Mrs. Amy Radcliffe, of Ambergate, who was Nurse Kean when the matters in question arose at the hospital, said Dr. Allen in no way instigated the writing of the letters of complaint. Miss Martin often had friends to visit her at the hospital.

In cross-examination witness admitted writing from Basford to Miss Martin, saying, "I write to humbly apologise for all I have done. I may say I never should have thought of doing such a thing if I had not been led into it by others. . . . I wish I had never had anything to do with the miserable affair. I told John Stone (the porter) I would not be mixed up in it at all, and he knows I was bothered to distraction to send in the complaints. . . . It was Nurse Perkins who started the mischief."

Witness: I did write that letter. I am sorry I did so.

The Lord Chief Justice: Do you represent that all that is untrue?

Witness: Yes, it is.

Mr. Atkinson: A tissue of falsehoods?—Yes.

Witness also admitted signing a statement, but said she had been worried into both these steps by plaintiff, who threatened to take steps which would cause her to lose her post.

Miss Athill, lady superintendent of the Royal Nurses' Institution, at Derby, stated that Miss Martin had been under her for some time, and she declined to allow nurses from the institution to work under Miss Martin.

### The Jury Disagree.

This concluded the case for the defence, and the Lord Chief Justice stated at once that he should hold that the occasion was privileged, and ask the jury whether there was malice.

After deliberating for over two hours, the jury sent a question to the Judge, and returned to court for his Lordship's direction.

It appeared that the jury were of opinion that the letter taken altogether was not malicious, but were divided on the question whether one portion of it had been actuated by malice. After a further deliberation lasting from 7.30 to 9.20, the jury returned, and the foreman announced that they had not agreed upon a verdict, and were as far from agreement as when they began.

The jury were then discharged.

### The Food Value of Milk.

By HELEN TODD, Matron, National Sanatorium, Bournemouth.

MILK has been termed a perfect food because all those elements which are necessary to sustain human life enter into its composition. Not only does it afford sufficient nourishment for the infant, but, under certain conditions adult life may be sustained upon it alone for long periods of time, and in many diseases the nurse must rely altogether upon milk for her patient's nourishment. It is therefore essential that she possesses the knowledge of what constitutes good milk and the ready means of testing its quality. The ignorance of many nurses in this respect is appalling, and unfortunately the effects of their want of knowledge may be most disastrous to their helpless patients.

Milk is "a compound fluid consisting chiefly of oleaginous and albuminous ingredients with water, sugar, and certain salts" (Hoblyn). The "oleaginous ingredients," consisting of minute fatty particles, are familiar to us in cream and butter; the "albuminous ingredients" we know chiefly as casein, which forms the principal constituent of cheese and to which skim milk owes the very considerable nutritive powers which it possesses.

Roughly speaking, the quality of milk is determined by the proportion of cream and the amount of solids contained in it, when of a certain specific gravity, and every nurse should know the simple methods of ascertaining these important points.

It is satisfactory to learn from so eminent an authority as Mr. E. Smith[1] that "fraudulent charges in milk are probably less than they ever were," but it is still necessary to be watchful for its adulteration by the addition of water or the abstraction of some part of its cream; these are details that can fortunately be fairly easily detected, although chemical adulterations require more complicated microscopic and chemical tests which can only be performed by the trained expert.

The public health authorities, as represented by the Public Analyst, recognising that milk varies very much in quality according to the breed of the cow, its food, and general condition, do not insist upon a very high standard. This is in order to avoid interfering unduly with the small retail dealer. They only require the cream to be not less than 9 per cent. of the volume of the milk, whereas a good average sample will show as much as 12 per cent., and milk from certain breeds of cows, such as Alderneys, will give us as cream 30 or even 40 per cent. of the whole volume of the milk.

The method of testing is as follows:—Take a glass cylindrical vessel graduated into 100 parts; fill it with the milk to be examined; let it stand undisturbed at a temperature of 60° Fahr. (15·5° C.) for eight hours, by which time it has been calculated that about 70 per cent. of the cream contained in the sample will have risen to the surface and any foreign bodies which were held in suspension at the time of the pouring of the milk into the vessel will have sunk to the bottom. Should the line of junction now visible between the cream and the rest of the milk be higher than the ninth division from the top marked on the glass the nurse may be certain that the milk has either been skimmed or deprived of some part of its cream.

The addition of a little water when the milk is first put into the glass vessel will cause the cream to rise much more rapidly, but if this be done it should be taken into account in the reckoning, and the specific gravity of the milk must first be ascertained.

Taking the specific gravity of milk is a useful test when in conjunction with that just described, but it cannot be relied upon alone, for milk being heavier and cream lighter than water, it is a very simple matter for the skilled adulterator to subtract a certain amount of the cream and then add water in the right proportion to produce the desired specific gravity, and so doubly detract from the nutritive powers of the milk.

[1] "Foods," Edward Smith.

The easiest method of ascertaining the specific gravity of milk is by means of the lactometer—a glass instrument consisting of a stem and bulb, the latter weighted with mercury and the former having a series of graduated figures marked upon it, ranging, from 1,000 to 1,040.

When this instrument is immersed in a vessel containing milk it will sink only so far as the specific gravity of the fluid will allow it, and this is shown by the figures which appear engraved on the stem at the surface of the milk.

It is important to pay attention to the temperature of the milk at the time of testing; thus the same sample[2] will be 1029 at 70° Fahr., 1030 at 60° Fahr., and 1031 at 40° Fahr. For purposes of comparison 60° Fahr. (15·5. C.) is taken as the standard temperature and 1030 to 1032 as the proper specific gravity of milk at that temperature. A too low specific gravity denotes a want of the nutritive solids which should be present; a too high specific gravity usually means a loss of cream.

The chemical reaction of milk, when tested with litmus paper, should be almost or entirely neutral. The presence of adulterants, such as chemicals, for the purpose of preserving the milk, can be frequently detected by this simple test.

A fourth method of estimating the food value of a sample of milk is by observing its opacity, for the poorer the milk the more transparent will it appear. For this purpose various instruments known as lactoscopes have been invented, the simplest being that known as "Fesler's" (of Munich). This consists of a cylindrical glass vessel having its upper part much wider than the lower part. A glass rod having dark lines engraved upon it is fixed into the narrower part of the tube. The milk to be examined is poured into the vessel until it reaches the junction of the tubes. The glass rod will not now be visible. The vessel is to be placed in a good light and water very slowly added to the milk until the dark lines can be clearly seen through the fluid. There are two sets of graduated figures and lines on the opposite sides of the upper part of the instrument, and the observer now notes those which have been reached by the milk and water; one set serve to show how much water has been necessary to make the dark lines visible, and the other to calculate the amount of cream in the sample.

[2] Parkes' "Hygiene and Public Health."

### THE NURSES' BOOKSHELF.

FIRST AID TO THE INJURED AND SICK. By DRS. WARWICK and TUNSTALL. (London: Simpkin Marshall. Bristol: Wright and Co. Illustrated. Price 2s. 6d.)

THIS book is profusely and well illustrated—an immense help to the beginner in ambulance work. It is perhaps open to the objection that simply and concisely as the subject of anatomy is dealt with, it yet goes rather far if its readers are non-professional. To the inexperienced, there is something so truly appalling in a sudden rush of blood for instance, that we fancy he would never have sufficient coolness to think out exactly the course of the wounded vessel, but content himself with knowing the simplest method of at least endeavouring to arrest the flow. However, knowledge never comes amiss, and the chapter on "The Blood and its Circulation" is most able and presented in an interesting form. The bandage section is largely illustrated, and with its aid ambulance classes will attempt and doubtless achieve great results. Chapter XV. on poisons is very useful and so arranged as to be understood by the meanest capacity. This is the more agreeable, as it is a subject generally somewhat overlooked in similar handbooks—a few antidotes being given to ordinary poisons, whilst others quite as common are not touched upon.

### County Council Lectures on Sick Nursing.

BY ONE OF THE LECTURERS.

It was in the autumn of 1898 that I was appointed sick nursing lecturer by the Technical Instruction Committee of one of our Northern County Councils. I had looked forward to such an appointment after some years of experience, and when the news came that I was a selected candidate for this appointment, with three others, I was in a state of great excitement.

### MEETING THE COMMITTEE.

It was arranged that we were to meet a portion of the Committee in the town forming the headquarters of the work, and in which the nurse appointed was expected to reside, during the time of lecturing. After a short journey I arrived there, and we were met by the organising secretary, under whom we were expected to work. I found only two candidates besides myself—the third having received an appointment and withdrawn—and during the time the Committee were assembling, we amused ourselves by discussing our various experiences in the work. One of the nurses had quite made up her mind that if appointed she must request leave to withdraw, as she could not possibly live in rooms by herself. The other would not really mind that at all—and so on went her rapid conversation, till when we could find time, we realised that we were in the presence of a "would-be" medical woman—a lecturer of six years standing, with a knowledge of human nature which could not be surpassed. My heart sank into my shoes as I thought of the hard work I had found my period of training, my knowledge of nursing only—though that I knew was thorough—and of my limited experience of lecturing, confined to delivering lectures on one or two occasions publicly, and on others to probationers in hospital. These reflections seemed to show that I had little chance against this brilliant candidate. And yet the idea occurred to me, would her nursing qualifications be what were expected? This somehow gave me courage, and I knew, if only I had the opportunity, how gladly I would and could carry my knowledge of sick nursing—learnt under some of the most admirable and painstaking of women in the nursing profession—to the humble cottage folk, whom it was hoped to help, and whom I have since learned are anxious to obtain that knowledge and help, aye, and use it when the time comes. My nursing experience carried the day and I was to commence duties shortly.

### MY FIRST LECTURE.

I have still very lively recollections of my first lecture in so public a capacity. It was in a country district several miles from a railway station, where I was to be met by a conveyance; but I was not very well up in the district, and instead of arranging a very early train in order to get to my destination for the afternoon lecture, I took it for granted I should get one about mid-day, which would give me ample time. On consulting a local guide, and finding the inconvenient condition of the local traffic, I realised that I had only a few minutes to catch the only train available for the journey—for me, at any rate. Tossing my notes, cap, and apron, etc., into a bag, I rushed to the station to find the train gone. "No other train on, lady, until 2.55," said the porter. "Better go round by C—— and drive from there." This I decided to do, at the same time knowing I should not arrive until after the time appointed, but I thought, "better late than never." Having arrived at C——, I hired a trap and a fast horse, and, with a very communicative driver, proceeded on my way. I think that man was a little shocked at my seeming inability to appreciate the scenery. Said he, "There's lots as signifies they could look for ever on this," as we passed the outskirts of a forest, glorious in its beauty of autumn tints. It certainly was a lovely view, and it would not have taken much to make him pull up on purpose to give me a better chance of admiring it. That we could not afford to do, so telling him that I was on most particular business, we proceeded. At last we arrived at the lecture-room, an hour late. The audience numbering 50 or so had partly dispersed; but on seeing me, those remaining reseated themselves, and after profuse apologies on my part to the local secretary, who did not receive my apologies with a very good grace, I proceeded to divest myself of my hat, etc.—as I was not wearing outdoor uniform—and arrange myself in my cap, apron, etc., for the purpose of giving my lecture. I was too disturbed to inquire whether there was any sort of dressing-room, and so I hastened to do this in the room, with the audience, mostly cottagers, looking on, and with an expression on their faces, which, had I been less worried, would have greatly amused me. I found the bedstead had been taken down again, as they had quite given me up; but it was brought back by two farm-labourers who had already packed it in a cart for removal, and with many half-smothered grumbles, and what sounded like stronger expressions of disgust, it was put up once more. After apologising to my audience, I endeavoured to proceed with the lecture, when to my horror I found that instead of the notes I had so carefully prepared, in order to prevent me from floundering until I got more accustomed to the work, I had put into the bag some other papers. However, pulling myself together, and feeling frightfully nervous, I began to hold forth on the duties of ventilation, cleanliness, bed-making, and so forth, in a way that, as I look back, under the circumstances, must have been fairly creditable. At any rate such overheard remarks as "Ain't she clever!" "Well it's quite natty" (referring to the bed-making), "And she's so sensible," impressed me that I had succeeded in gaining their confidence. I have had many lectures in that room since, but I always see myself on my first arrival and live over again that one and a half hour, just for a few moments, at every visit.

### AN AUDIENCE OF 600 PEOPLE.

I have had various audiences up and down the county; some small, others large, some most enthusiastic, others shy though in most cases a very fair amount of interest has been shown, but my "great" ovation was my first appearance in a small manufacturing town. Previously, I had lectured to 100 and 200 in the same district so that I was not quite so awed as I might have been. The course to be given was the "First," containing lectures on bed- and poultice-making, treatment of cuts, etc., and the management of children, and they were given in an admirable room, with a platform running along one side. We had a most energetic local secretary, and before my appearance on the platform I was informed by him that there were "a good number" awaiting me, but when I saw the good number I was quite overjoyed. It seemed so encouraging that so many people in one small town could be found to take an interest in nursing the sick. I was received with a burst of applause, which was also new to me, but it pleased me; I knew the people, and that their welcome was genuine, also that they did not mind showing it. I was nervous as to whether I was making myself heard at the other end of the room, and after the lecture I inquired of one or two motherly looking women, whom I had noticed in the gallery, as to whether they had any difficulty. "Bless yer! noa," said one. "Yer voice ain't of the loudest, but it do carry. Yer couldna tell mony secrets." That was a relief, although I

got the information so bluntly. I had invited any of the people who liked to put questions to me at the close of the lecture, so the platform was besieged, and those who did not ask questions came to shake hands, for, as I overheard, "She be worth it! If everybody looked as well as her there would be nowt wrong with us!" I have had many such experiences of this kind and many warm receptions, and I have also had the gratification of learning of the improvements in "Home Nursing" after the lectures had been given in the district. This has amply repaid me for the trouble taken and for the wear and tear of continual travelling. Lives have been saved by the knowledge of "first-aid" treatment, and illnesses averted by the timely and proper application of a poultice.

### Nursing in Manila.

By a Correspondent.

The urgent need of a civil hospital was proposed by the board of trustees to Mrs. Whitelaw Reid with the result that she forwarded $3,000 to be used as the managers directed. Very soon a suitable house was chosen, situated on Gral Salano, in San Miguel, just above the Ayala Bridge, making the hospital accessible both by land and water. It was temporarily equipped by the Military department while waiting shipment of furniture and goods from the United States. The operating room was completely furnished through the generosity of Colonel Greenleaf, Surgeon-General to the American Army in the Philippines, and many others gave valued contributions.

The institution is named "The Women's Hospital," but, being the only place of its kind in Manila, the trustees have decided for the present to admit men in need of special care. Major McDill, army surgeon, is President of the House Committee. There is a board of twelve consulting physicians and surgeons chosen from the leading practitioners in Manila, both native and American. The lady patronesses are about 50 in number.

The superintendent of nurses, Miss Mary E. McDonald, is a graduate of the Belle Vue Training School for Nurses. She has enjoyed a wide experience both in private and in military hospitals, and her assistants are fully-trained and graduate nurses from various schools and hospitals, namely, Miss Mary L. McCormick, Erie County Hospital, Buffalo; Miss Effie Wolfe, Illinois Training School for Nurses; Miss Louise M. Mount, Long Islands College, N.Y.; Miss Mary G. Barker, Roosevelt Hospital, N.Y. The supervisor of the diet kitchen is Miss Mary A. Welsh, from the Grand Rapids Training School for Nurses. These ladies are assisted by a male nurse from the Hospital Corps and twelve natives.

The hospital, though founded by subscriptions, will be self-supporting, and the patients are required to pay according to their means; those unable to pay for a private room can be placed in a room with two or three beds. The patients are allowed to choose their own physicians and surgeons, and free patients are attended by one of the consulting staff. Any doctor is allowed the use of the operating room on payment of a small fee for any operations which do not necessitate the patients remaining in the hospital. In treating cases in the hospital, every doctor is a free and independent agent, as much so as if attending cases in their own homes. The hospital has a directory for nurses, by means of which, or by telephone, any available nurse in private practice and not engaged with a case will be sent where she is wanted.

Private nursing in Manila is on the American system. Each nurse, on receiving her diploma, starts a practice. If incompetent, or addicted to habits which bring discredit on the profession, neither doctors nor patients will employ her, and of necessity she is obliged to choose some other means of earning a living. But it is no more difficult to get on and succeed than elsewhere provided that the nurse be competent and desirable.

### Resignation of Miss Griffiths.

In last week's Nursing Mirror we alluded to the resignation of Miss Griffiths, for many years matron of Lambeth Infirmary. The high appreciation in which her work is held by the Guardians is shown by the following letter, addressed to Miss Griffiths, and read at the meeting of the Board on Wednesday (7th inst):—

"Dear Miss Griffiths,—The Guardians have heard with profound regret of your intention to relinguish your office of matron of their infirmary in October next, and they are more especially sorry that ill-health is the cause of your leaving. They feel, however, that it will be some satisfaction to you to know how highly they appreciate your constant endeavour to render efficient the nursing staff under your charge, to maintain a high order of discipline, and to see that due care and attention are bestowed on the sick and suffering. The Board, more especially the older members, are aware of the many difficulties with which you have had to contend during your twenty-eight years of service as matron, and of the unswerving and unflinching manner in which you have ever followed the path of duty which conscience and a thorough knowledge of the requirements of the institution have dictated to you. It must, indeed, be a source of gratification to you to look back on the long years of trial, of difficulty, of hard work, and of constant strain, and to know that from all these has resulted in a large measure the good order and efficiency which now exists. The work has brought its own reward in the esteem in which you are held alike by the Guardians and the officers with whom you are brought in contact. As a mark of the appreciation of the Guardians of the services you have rendered to the parish, it is their intention, unanimously expressed at their last meeting, to apply to the Local Government Board for permission to increase your retiring allowance to the maximum allowed by the Poor Law Officers' Superannuation Act, and it is their earnest hope that you may be spared to enjoy many years of quiet content after your long and honourable official career."

### Appointments.

Huntingdon County Hospital.—Miss Neve has been appointed nurse matron. She was trained at the London Hospital, and has since been matron at Enfield Cottage Hospital, Blandford Cottage Hospital, and the Royal Cornwall Infirmary.

Mill Lane Fever Hospital, Liscard, Cheshire.—Miss Caroline Terrey has been appointed charge night nurse. She was trained at the Royal Southern Hospital, Liverpool, and has since been charge nurse at the Royal National Hospital, Ventnor; on the private staff of the Royal United Hospital, Bath; charge nurse at the Northern Fever Hospital, Winchmore Hill; nurse at the Park Fever Hospital, Hither Green, Lewisham; and, from September last, charge nurse at the Ham Green Hospital, Bristol.

Poole Workhouse Infirmary.—Miss J. M. Cooper has been appointed superintendent nurse. She was trained at the Chorlton Hospital, Manchester, where she has since held the posts of charge nurse and midwife.

Sheffield Union Infirmary.—Miss Annie Hunt has been appointed maternity charge nurse. She was trained at Walsall Union Infirmary, and has since been trained ward sister at Bradford Union Infirmary.

St. Mary's, Islington, Infirmary.—Miss M. L. Brentnall has been appointed staff nurse. She was trained at Chorlton Union Infirmary, and has since had charge of sick wards at Hinckley Infirmary.

### Echoes from the Outside World.

It seems such a short time since I wrote about the funeral of our own dear Queen. Now it is the last obsequies of her eldest daughter which occupy the minds alike of English and German women. As in the case of the Duke of Saxe-Coburg, the removal of the body from the home to the church was carried out at night, the first sound of muffled drums, announcing that the departure was imminent, being heard at half-past nine on Saturday evening. The funeral procession was fitly formed beneath the porch at Friedrichshof, which bears in Latin the words, "To the Memory of Frederick." The spacious square in front of the house was lined with soldiers, standing shoulder to shoulder, and carrying in their hands flaming torches, and the route to the small church—where the Empress Frederick so often worshipped—was guarded in the same manner, making a most impressive and awesome ceremonial in the darkness around. The coffin was borne upon the shoulders of ten soldiers, and immediately behind walked the Kaiser, her late Majesty's sons-in-law, Prince Christian of Schleswig-Holstein, and his son, and many other royal personages. The King of England did not arrive in time to take part in the first ceremony, and no ladies were present. Before the German Emperor left the body in front of the altar he knelt down in prayer for a few minutes, evidently deeply affected, and ere he turned away he bent forward and kissed the coffin.

On Sunday afternoon a special service was held in the church at Cronberg, which was attended, in addition to the other royal persons, by King Edward, looking tired and sorrowful, and by Queen Alexandra, who showed less weariness and appeared more calm. The interment itself took place at the Potsdam Mausoleum on Tuesday morning, and the ceremony was entirely military, none but the royal relatives being allowed to enter the last resting of one who was deeply beloved whilst she remained upon earth, and who has left behind her much of her power for good. At an address given in the English church at Homburg by Canon Teignmouth Shore, the preacher, who was present at the last moments, repeated a touching little incident. He said, "A white butterfly—ever recognised as the emblem of the Resurrection—rested for a moment on the dying Empress and then flew off through the open window out into the light and soared towards the blue—meet type it seemed, indeed, of that pure soul departing and shaking from its white wings the dusts and dews of mortality." Many appropriate references were made to the illustrious lady in English pulpits on Sunday, and the general mourning for three weeks, which has been ordered by the King, will be a suitable visible indication of the feelings of the people among whom her early years were spent.

The new proclamation issued by Lord Kitchener ought to commend itself to the commonsense of the country. It is concise and determined in tone, without being harsh or unjust, and even the least sanguine are hopeful that it may have a good effect upon the poor misguided Boers, unless the commandoes are successful in keeping from their men any knowledge of its existence. This, of course, they will endeavour to do so as to prolong the warfare, but as Lord Kitchener is sure to circulate the terms of his declaration amongst the women in the concentration camps, they will doubtless spread the news amongst their relatives. The wives will naturally bring all their influence to bear, because unless "all commandants, field-cornets, and leaders of armed bands still engaged in resisting His Majesty's forces" surrender before September 15 they will be permanently banished from South Africa. And in addition, "the cost of the maintenance of the families of all burghers in the field will be recoverable from such burgher and will be a charge upon their property movable and immovable in the two colonies." The proclamation has evidently been the outcome of much consultation and deliberation, for it was framed when Lord Milner was here, the Cape Government entirely concur, and Lord Kitchener approves. That it may be the means of putting "an end to a state of things which is aimlessly prolonging bloodshed and destruction and inflicting ruin upon the great majority of the inhabitants," as the proclamation says, is, I am sure, the fervent wish of every nurse and every woman in the country.

It has been suggested that if Prince Henry of Orleans, who has been cut off in the flower of his manhood, had lived some years longer he might have been a dangerous enemy to England. In any case it is a waste of time to speculate on any thing that might have been; and to choose the morrow of the young man's death for dwelling on his potentialities for evil if his career had not been prematurely terminated by fever contracted in the Far East does not strike one as generous. It is more pleasant to bear in mind the fact that, however mistaken he was in his views about England, his fidelity to his native country remained unshaken, though his native country would not allow him to serve her because he happened to be a prince of the blood-royal. There is no doubt that he was born with the love of adventure, and, denied it in one way, he did his best to console himself for exclusion from the French army by perilous explorations in Tibet and Abyssinia, by a quarrel with his friend and comrade, M. Bonvalot, and by a duel with Prince Victor of Italy. Happily for his own reputation, his enmity to England—which was all the more remarkable because he was born in Middlesex, and was always treated with the utmost courtesy here—did not prompt him to descend to the scurrilities which have rendered another scion of the house an object of contempt.

How good it is just now to leave the city behind and fly to the country and the sea! The heat of last week seems to have driven forth many long-suffering citizens, and it was most delightful to find ourselves spinning away from Waterloo as fast as a capital train would carry us to Axminster, en route for Charmouth. We had made jokes as we came along that we would purchase a carpet cheap on the spot, only to find upon inquiry that all Axminster carpets are made at Wilton to which place the industry was removed, nearly seventy years ago. As a distinct contrast, Axminster now principally occupies itself in mending lace. As far as we were concerned, Axminster was the end of our railway journey, for we had to reach Charmouth by coach. Next year a light railway will, it is hoped, be opened in the spring, which will of course be convenient for the saving of time, but I would not have missed that beautiful six miles ride for anything. As we mounted higher and higher, fresh beauties of green pastures, waving cornfields, wood-crowned summits, far-reaching moors, and heather heights were disclosed to view, the air became bright and light like champagne, and I felt as if I must keep opening my mouth to drink in the invigorating draught. Charmouth itself is most quaint. The village begins at the top of a high hill, and straggles gently down in one long line till it reaches the bottom. In the centre of the street is a house in which Charles II. took shelter whilst trying to escape from Dorsetshire after the battle of Worcester, to France, and the Danes landed at Charmouth in the days of old and many times fought the Britons there. The accommodation now, though limited, is good, clean, and moderate, the sea-shore is very picturesque, and the bathing good, whilst the walks around are sufficiently numerous and varied to allow of a fresh one every day for a week or two.

### Everybody's Opinion.

[Correspondence on all subjects is invited, but we cannot in any way be responsible for the opinions expressed by our correspondents. No communication can be entertained if the name and address of the correspondent is not given, as a guarantee of good faith but not necessarily for publication. or unless one side of the paper only is written on.]

### THE NURSES OF THE METROPOLITAN HOSPITAL.

"MISS E. C. KINGSFORD" writes from Brondesbury: In the interesting chat between your commissioner and the matron of the Metropolitan Hospital, Miss Bennett, I am sure, quite unintentionally gives your readers a wrong impression as to the initiation of the new *régime* at that institution. The alterations in the condition of the nursing did not begin when Miss Cave, her predecessor, was matron, but they were started and carried out, as far as time would permit, by Miss Cave's predecessor Miss Kingsford; and it was entirely on account of the extra strain and worry involved in the reorganisation of the nursing scheme that Miss Kingsford's health unhappily broke down early in 1897. I note this fact partly as a historic correction and partly because I feel sure that Miss Cave would be most unwilling to take credit for work that was done by another. While on the subject I should like to say a word as to the salary paid to private nurses connected with the Metropolitan Hospital. There is an article, I observe, in your current number on the subject of nurses' fees, and in many respects I do not at all agree with its writer, for I suppose that we all admit that "the labourer is worthy of his hire," and we ought, as Dr. Goodhart so aptly put it the other day, "to see to it that the hire is worthy of the labourer." Now I maintain that a woman who has given up three or four years of her life to the labours and risks of hospital work is fully entitled to at least the market value of her subsequent services; and seeing that a trained nurse may earn as much as £100 per annum, and actually does earn from £50 to £80—to put it at a low computation—why should the middleman, the nursing home or hospital as the case may be, pay her the inadequate salary of £28, and secure the surplus for his own use? In the case of a nursing home or institution a small percentage should be deducted from each nurse's earnings for the upkeep of the establishment on the plan first instituted by the Nurses' Co-operative Society; but no hospital is justified in exploiting its nurses for the benefit of its own coffers. And I sincerely trust that, when Miss Bennett's dream is realised and the Metropolitan Hospital has its own nursing home, those in authority will "see to it that the hire is worthy of the labourer."

### SPITTOONS AND DISINFECTANTS.

"NURSE GIRTHA" writes: I was greatly interested in reading Dr. Dalton's lecture on pneumonia and also the letter of a matron who had been present at the British Congress on Tuberculosis. Now I should imagine that every trained nurse knows how imperative it is that all spittoons should have disinfectants at the bottom (Dr. Dalton advises 1·20 carbolic acid); but does every nurse know that if Sanitas be used, the expectoration on coming in contact changes its colour to a "rusty brown," the unfavourable symptom in croupous pneumonia? When working in private houses it is not always possible to have all one wants, and I have found that permanganate of potash and Sanitas are the most general disinfectants to be found. It is of course impossible to use the former when the sputum is to be examined, but the latter being colourless is usually thought to answer the purpose. But it is very important to remember this point. In my own experience I always inform the patient's medical adviser, and in one instance the doctor, a well known man, absolutely refused to accept the statement. But he was eventually convinced after watching the sputum carefully for several days—first with and then without the addition of Sanitas.

### SECTARIANISM IN NURSING.

"A. M. B." writes: It would indeed be well if the justice-loving British public were made acquainted with the persecution which many Roman Catholic nurses undergo on account of their religious views—that, too, in well-known hospitals which pride themselves upon being unsectarian. At one institution in particular which occurs to my mind Catholic nurses were compelled to attend Protestant prayers or to suffer the alternative of immediate dismissal. They consulted the rector of their parish, who called upon the matron. She told him that the rule had been made by the Board. When the Board was consulted each member denied all knowledge of the rule, even saying that the matter was a private one for the nurses' consideration and entirely beyond their jurisdiction. They did not interfere, however, and the nurses were compelled to submit to this tyrannous and despotic state of things. They had to listen to insulting personal remarks at the public dinner table from the matron. In fact, by numerous petty persecutions their life in the hospital was rendered well-nigh unbearable. They were not "requested to resign," the *modus operandi* was not characterised by such straightforwardness, but with one exception they eventually did so. The remaining nurse was ultimately dismissed upon a ridiculous pretext. Another nurse's cap-strings were found among her laundry articles which she had just put away, and upon being practically accused of theft by the lady superintendent she quite politely demanded an apology. Whereupon she was told to resign at once or be dismissed for "insubordination." In this hospital nearly every other religious sect is represented on the staff, and neither the matron nor the lady superintendent is a member of the Established Church. There are also one or two Agnostics and Atheists, but their private views are not interfered with. Why does this state of affairs exist? It is particularly sad when one reflects that it occurs in a body of Christian women banded together to do a noble work and all working for the same cause.

### NURSES WHO SHOULD BE WEEDED OUT OF THE PROFESSION.

"ANOTHER SUFFERER" writes: Seeing the letter "Nurses who should be weeded out of the Profession," I, too, would ask the question, Can nothing be done to prevent such nurses remaining in the profession? I fully endorse every word of "Matron's" admirable letter. Testimonials and references are only too often not worth the paper they are written on. This is a training school for midwifery, and several times I have dismissed pupils on account of intemperance. To my chagrin I have later on seen their names among those of the successful candidates for the L.O.S. certificate; and yet the society requires a certificate of "moral" character. I quite agree that they are often very clever women, but the injury they do a Home in one month takes months to recover from. There are too many women who take up nursing because they think there is not hard work attached to it, and after leaving their hospital take care that they do not do more than they can possibly help. They join a nursing home with a fixed salary, and then do not care how their work is done. The public are very much to blame, for they do not like to report a nurse for fear of her being dismissed, but in future they do not send to that Home for a nurse. I know of several instances, not only in connection with my Home, but with other and larger institutions where this has happened. How is a matron to know when a nurse is not giving satisfaction, if the patient or friends will not make a complaint? I only recently have had too great a cause for complaint. Being hastily summoned away and unable to return for a month I left my assistant, who is a trained nurse and midwife, L.O.S., in charge, who during my presence was satisfactory. She wrote regularly each week, "All going on well in home and district." I returned home to find everything neglected, patients and pupils discontented, and yet they would not write and tell me how things were because, as they put it, "It's not your fault and you had worry and anxiety enough;" but the result is the same—the patients have gone home because they could not get proper attention, and whereas I went away leaving over 60 patients on my books, on my return they were reduced to 25; and it is only after a month's hard work night and day that things are getting right again. Needless to say I gave the assistant notice. Yet she came to me with excellent testimonials, which she will doubtless use to get another post. I may say that drink was not this nurse's failing, but laziness.

### For Reading to the Sick.

### THE GREAT ESSENTIAL.

I ASK Thee for a thoughtful love,
Through constant watching wise,
To meet the glad with joyful smiles
And wipe the weeping eyes;
And a heart at leisure from itself
To soothe and sympathise.

May I reach
That purest heaven,—be to other souls
The cup of strength in some great agony!—
Enkindle generous ardour,—feed pure love,—
Beget the smiles that have no cruelty,—
Be the sweet presence of a good diffused,
And in diffusion ever more intense!
So shall I join the choir invisible,
Whose music is the gladness of the world!
*G. Eliot.*

O Thou, Who keep'st the Key of LOVE,
Open Thy fount, Eternal Dove,
And overflow this heart of mine,
Enlarging as it fills with THEE,
Till in one blaze of charity
Care and remorse are lost, like motes in light divine!
Till, as each moment wafts us higher,—
By every gush of pure desire,
And high-breath'd hope of joys above,
By every secret sigh we heave,
Whole years of folly we outlive,
In His unerring sight Who measures Life by LOVE.
*Keble.*

Ask Him to increase your powers of sympathy: to give you more quickness and depth of sympathy, in little things as well as great. Opportunities of doing a kindness are often lost from mere want of thought. Half a dozen lines of kindness may bring sunshine into the whole day of some sick person. Think of the pleasure you might give to some one who is much shut up, and who has fewer pleasures than you have, by sharing with her some little comfort or enjoyment that you have learnt to look upon as a necessary of life—the pleasant drive, the new book, flowers from the country, etc. Try to put yourself in another's place. Ask "What should I like myself, if I were hard-worked, or sick, or lonely?" Cultivate the habit of sympathy.
*G. H. Wilkinson.*

Let every creature have your love. Love, with its fruits of meekness, patience, and humility, is all that we can wish for to ourselves, and our fellow-creatures; for this is to live in God, united to Him, both for time and eternity. To desire to communicate good to every creature, in the degree we can, and it is capable of receiving from us, is a divine temper; for thus God stands unchangeably disposed towards the whole creation.—*Wm. Law.*

"The Everlasting Arms." I think of that whenever rest is sweet. How the whole earth and the strength of it, that is almightiness, is beneath every tired creature to give it rest; holding us, always! No thought of God is closer than that. No human tenderness of patience is greater than that which gathers in its arms a little child, and holds it heedless of weariness. And He fills the great earth, and all upon it, with this unseen force of His love, that never forgets or exhausts itself, so that everywhere we may lie down in His bosom, and be comforted.—*A. D. T. Whitney.*

### Notes and Queries.

**The Editor is always willing to answer in this column, without any fee, all reasonable questions, as soon as possible.**
**But the following rules must be carefully observed:—**
1. **Every communication must be accompanied by the name and address of the writer.**
2. **The question must always bear upon nursing, directly or indirectly.**

**If an answer is required by letter a fee of half-a-crown must be enclosed with the note containing the inquiry.**

### *Holiday Home for Nurses.*

(192) Will you kindly tell me if there is a hostel or holiday home for nurses in Edinburgh or Glasgow?—*N. A.*

Perhaps a reader can recommend one. Holiday homes are outside the range of nursing, and particulars are not easy to obtain.

### *Heart Specialist.*

(193) Will you kindly give me the name of a heart specialist, and tell me what his fee is, and also if he would make any reduction to a nurse.—*A. S.*

We cannot recommend specialists. A specialist's fee is usually £2 2s. for the first consultation, and £1 1s. per visit after.

### *Dispensing.*

(194) I wish to pass the examinations of the Pharmaceutical Society later on; but, having a little spare time now, I wish to know if I could prepare some of the subjects privately, or join a correspondence class?—*E. R.*

You should write direct to the Secretary of the Pharmaceutical Society, 17 Bloomsbury Square, W.C., as he may be able to direct your course of studies to the greatest advantage.

### *America.*

(195) I am anxious for work in Canada, or the United States. I am a Queen's Nurse, and should prefer district to private work. Can you tell m to whom I should apply for information?—*Nurse Elain.*

Apply the Lady Superintendent, the Victorian Order of Nurses for Canada 578 Somerset Street, Ottawa, Ontario, Canada.

### *Eczema.*

(196) A young woman, who has to earn her own living, is terribly troubled with eczema. The remedies prescribed by the local doctor, who has seen her thrice, have done her no good, and she is now trying patent medicine and bathing it with whiskey. After reading the article on eczema in the HOSPITAL I feel sure that something could be done for her if one only knew what. 2. Is it any advantage to a provincial nurse to have her name and address in "Who's Who"?—*Monthly Nurse.*

Consult a specialist. 2. If you mean in "Burdett's Official Nursing Directory," decidedly.

### *Case for Massage.*

(197) Owing to a serious accident a young man is unable to use his leg. The doctors are of opinion that a thorough course of massage is necessary. Is there any institution where he could receive it free, or, for a small payment?—*District Nurse.*

You might write and ask if the case is suitable for admission into the National Hospital for the Paralysed and Epileptic, Queen's Square, Bloomsbury, W.C.

### *Colonial Nursing Association.*

(198) Will you kindly give me the address of the Colonial Nursing Association; and also those of any other agencies for obtaining hospital appointments abroad?—*Sister Agnes.*

The Imperial Institute, S.W. There is no other agency especially for this class of appointment.

### *Spinal Case.*

(199) Can you give me addresses of any homes suitable for a little boy of four suffering from spinal complaint. He is not considered a suitable case for a hospital, and his parents can only afford 2s. a week for him.—*N.E.*

Apply Cheyne Hospital, Cheyne Walk, Chelsea, S.W.; The Hospital and Home for Incurable Children, 2 Maida Vale, W.; and the Hospital of St. John and St. Elizabeth, St. John's Wood, N.W.

### *Baden-Powell's Nurses.*

(200) Would you kindly tell me the correct channel for application for nurses wishing to join Major-General Baden-Powell's Nursing Staff? Is there an office in London?—*Sister Eleanor.*

The vacancies on this staff are filled up locally.

### *Substitute for Cream.*

(201) If an infant require the addition of cream to its daily food, and it is unprocurable on account of expense, would it be safe to add fresh beef suet to the milk instead?—*B. C.*

Fresh beef suet tied up in a muslin bag and boiled in the milk will make it a little more fatty, but it is a poor substitute for the natural cream of good milk.

**Standard Books of Reference.**

**"The Nursing Profession: How and Where to Train." 2s. net; post free 2s. 4d.**
**"Burdett's Official Nursing Directory." 3s. net.; post free, 3s. 4d.**
**"Burdett's Hospitals and Charities." 5s.**
**"The Nurses' Dictionary of Medical Terms." 2s.**
**"Burdett's Series of Nursing Text-Books." 1s. each.**
**"A Handbook for Nurses." (Illustrated.) 5s.**
**"Nursing: Its Theory and Practice." New Edition. 3s. 6d.**
**"Helps in Sickness and to Health." Fifteenth Thousand. 5s.**
**"The Physiological Feeding of Infants." 1s.**
**"The Physiological Nursery Chart." 1s.; post free, 1s. 3d.**
**"Hospital Expenditure: The Commissariat." 2s. 6d.**

**All these are published by THE SCIENTIFIC PRESS, Ltd., and may be obtained through any bookseller or direct from the publishers, 28 and 29 Southampton Street, London, W.C.**

### Travel Notes.

BY OUR TRAVELLING CORRESPONDENT.

### LXXIX.—IN THE VALLEY OF THE TAUNUS.

THE Taunus country is richly wooded and is an excellent locality for those who like good walking combined with delightful forest scenery. It is now chiefly visited however by those in search of health, as dispensed at the numerous natural springs, known and appreciated as long ago as the days of Pliny.

### THE CHIEF HEALTH RESORTS.

I think the best known are decidedly Wiesbaden, Langen, Schwalbach, and Schlangenbad. Of Wiesbaden I must not stay to say much, for it is well known to the travelling public. The springs are chiefly useful in diseases of the respiratory organs—gout, neuralgia, and rheumatism, and there are splendid establishments organised for the benefit of the poor. Wiesbaden is, however, so gay a place that somehow one overlooks the fact that it is a health resort. The gay crowds that throng its promenades, visit the Kursaal, and listen to the bands seem to have no connection with health seeking.

### THE COST OF THE JOURNEY.

For those contemplating a serious stay at one of the well-known spas, and whose means are limited, I will give some particulars. I will quote the expense of the journey to Wiesbaden—Schwalbach and Schlangenbad being a short distance further.

First-class return to Wiesbaden for thirty days £4 9s. 8d., second-class £3 1s. 4d. (N.B.—I never travel first-class myself.) The 30 days can be extended by an arrangement beforehand.

It is well to consider carefully, where you mean to take your after-cure, as that would probably mean returning by a different route. After a course of treatment at Schwalbach, for instance, it is necessary to go for a fortnight, or a month if possible, to some bracing climate—the Swiss mountains, or the higher parts of the Tyrol or Black Forest—unless your home should chance to be in the elevated districts of the Lake Country or Scotland.

The journey from London to Schwalbach takes 24 hours, and can be done easily without a break; there is a rest of more than two hours at Cologne, which gives time for a comfortable wash, a good lunch, and a visit to the cathedral, which is close to the station. Last year having experienced an extremely bad crossing to the Hook, and feeling in consequence somewhat battered, I fell sound asleep in the magnificent Dom, and was only awakened by the magnetic properties of a pair of astonished and scornful eyes fixed upon me, belonging to a very correct Englishman, who, properly armed with his Baedeker and looking the very incarnation of British respectability was conscientiously studying the wondrous pile.

### LIFE AT SCHWALBACH.

It is not an expensive cure and small means need not deter you from trying it. There are several first-class hotels, and even these are reasonable compared to prices at Homburg and Carlsbad, but there are plenty of pensions where you will be very comfortable for 6 or 7 marks a day, or even less.

The doctor's fees are very moderate, and my experience of them is, that they are most considerate and kind and do not pay unnecessary visits. The usual routine is, water drinking at 7 or 7.30 A.M., breakfast at 8.30 or 9. Bath at 10 or 11, according as hours fit in, for in the height of the season it is somewhat difficult to arrange for all; then another glass of water taken whilst gently walking about. Lunch, abundant but simple, at 1 o'clock, then reclining on *chaises longues* under the trees (an amusement which occupies all stray moments, as much walking is discouraged). At 3 P.M. another glass of water, at 5 coffee at a good confectioner's above the gardens; more *chaise longue*, or perhaps a very mild walk. Supper at 7.30, and, if the weather is propitious, another hour in the gardens to listen to the band; or if it rains an adjournment to the Kursaal for a concert or dancing. All Schwalbach is in bed and in slumber wrapt by 10 or 10.30. The cure is chiefly for those suffering from anæmia of all sorts and kinds, debility of the mucous membranes and catarrhal affections, and the results are surprising. There is a large quantity of iron in the water, it is assimilated most remarkably, and those who are unable or imagine themselves unable, to stand iron in medicine, absorb it unquestioningly here and soon reap the benefit.

The waters taste rather like flat soda-water and are sucked through glass tubes whilst the drinker slowly promenades the gardens and listens to the band, which plays three times a day to encourage the victims!

It is particularly advantageous to be as much in the open air as possible, so that those determined to gain all the benefit possible lie out on their *chaises longues* all day, in spite of cold or even of rain, if not too heavy. The effect is very comical of seeing (on a cold day) numberless motionless mummies extended under the trees, waited on by family slaves as represented by attendant husbands, brothers, etc.

At 3 P.M. the mummies unwind themselves, and assuming the perpendicular adjourn to the various springs for their portion of water.

*(To be continued.)*

---

### TRAVEL NOTES AND QUERIES.

RULES IN REGARD TO CORRESPONDENCE FOR THIS SECTION.—All questioners must use a pseudonym for publication, but the communication must also bear the writer's own name and address as well, which will be regarded as confidential. All such communications to be addressed "Travel Editor, 'Nursing Mirror,' 28 Southampton Street, Strand." No charge will be made for inserting and answering questions in the inquiry column, and all will be answered in rotation as space permits. If an answer by letter is required, a stamped and addressed envelope must be enclosed, together with 2s. 6d., which fee will be devoted to the objects of the "Hospital" Convalescent Fund. Any inquiries reaching the office after Monday cannot be answered in "The Mirror" of the current week.

PARAMÉ (Pedestrian).—It is not quite my idea of the place for you. True, the sea is fine, but it is so completely a place of hotels and lodging houses. As expense does not seem a primary consideration, why not make Dinard your head quarters. The walks are beautiful, especially in the direction of Dinan, and by crossing the bay (time occupied, 15 minutes) you can explore all the country on the other side of the Rance. St. Suliac is a charming spot. I fancy Dinard would be far preferable for your companion, there is so much in the place itself for non-walkers. The two bays for bathing are unsurpassed, and the steam ferry that connects St. Malo, St. Servan, Paramé, and Dinard every half hour makes constant change without fatigue possible.

ROME FOR THE WINTER (Clericus).—There is no better book for your purpose than "Walks in Rome," by Hare. It gives you all necessary information, and much besides, even to the best hours for making sketches. You would also be interested, if you do not know them, in Mrs. Elliott's "Roman Gossip" and Marion Crawford's "Ave Immortalis." You probably know Hawthorne's "Transformation," though it is now out of fashion, and Ouida's "Ariadne."

LEITH TO ROTTERDAM (Nurse Mary).—The lack of language would not much matter, but Holland is not cheap. You could live in Belgium for 5s. per day comfortably, and the Ardennes is charming (Southern Belgium). To reach that you could go from Leith to Antwerp. I cannot find that Grangemouth steamers go to Rotterdam, but it may be so. I do not think I could recommend you anything so cheap as 5s. per day in Rotterdam. If it is not too late, let me hear again more definitely.

ANTWERP OR HAMBURG (Reta).—I find the Tower Bridge boats no longer run to Antwerp and Hamburg, but the G.S.N.C. starts for those ports viâ Harwich. Fares to Antwerp, first return, £2; second, £1 4s. Fares to Hamburg, first return, £2 16s. 3d.; second, £1 18s. 9d. But if you can really only stay a very few days, these distances are too great—you would be all the time in the boat. It takes about 20 hours to Antwerp, and something like 25 to Hamburg. If you decide to go to Antwerp, ask for a room on the third floor at the Hôtel du Commerce, Rue de la Bourse. Your room, breakfast, and dinner, will come to about 6 francs, that is 5s. Or if that is full, try Hôtel Fleur d'Or, 1 Ruella des Moines. At Hamburg, Hôtel Frankfurt, 8 Bergedorfer-Strasse, or Hôtel Kaiserhof, 11 Bahnhof-Strasse, both about the same terms. I am sure you will make a mistake if you go so far. Why not take the Tower Bridge boat to Ostend? Three times a week; fares: first return, 10s. 6d.; second, 9s. On all these boats second is quite comfortable; but where the difference is so small, as to Ostend, I should go first. Then go to Knokke, Hôtel de Bruges, very reasonable; you might stay a week for about 27s. or 30s. Send stamps to manager, and ask for my article on Knokke, of September 1st, 1900. It will give you all particulars.

